# Stem cells for the treatment of early to moderate osteoarthritis of the knee: a systematic review

**DOI:** 10.1186/s40634-023-00665-1

**Published:** 2023-10-07

**Authors:** Theofylaktos Kyriakidis, Charalampos Pitsilos, Myrto Iosifidou, Alexandros Tzaveas, Ioannis Gigis, Konstantinos Ditsios, Michael Iosifidis

**Affiliations:** 1 Department of Orthopaedic Surgery and Traumatology, Erasme University Hospital, Université Libre de Bruxelles, Route de Lennik 808, 1070 Brussels, Belgium; 2 2nd Department of Orthopaedic Surgery and Traumatology, Aristotle University of Thessaloniki, “G. Gennimatas” General Hospital, Ethnikis Aminis 41, 54635 Thessaloniki, Hellas Greece; 3 3rd Orthopaedic Department, Interbalkan Medical Center, Thessaloniki, Greece; 4 Orthobiology Surgery Center, Thessaloniki, Greece

**Keywords:** Knee osteoarthritis, Outcomes, PROMs, Mesenchymal stem cells, MSCs, Regenerative medicine, Intraarticular injection

## Abstract

**Purpose:**

Mesenchymal stem cells (MSCs) present a valuable treatment option for knee osteoarthritis with promising results. The purpose of the present study was to systematically review the clinical and functional outcomes following mesenchymal stem cell application focusing on early to moderate knee osteoarthritis.

**Methods:**

A systematic search was done using the Preferred Reporting Items for Systematic Reviews and Meta-Analyses guidelines in Pubmed, Scopus, Web of Science, and Cochrane Library databases. All Studies published between 2017 and March 2023 on patients treated with single mesenchymal stem cell injection for Kellgren-Lawrence grade I—III knee osteoarthritis reported on clinical and functional outcomes were included.

**Results:**

Twelve articles comprising 539 patients and 576 knees treated with a single intraarticular injection of MSCs for knee osteoarthritis were included in the current systematic review. In eligible studies, the reported outcomes were improved concerning patient-reported outcomes measures, knee function, pain relief, and quality of patient's life.

**Conclusion:**

Based on high-level evidence studies, single intraarticular injection of MSCs is a safe, reliable, and effective treatment option for Kellgren-Lawrence grade I—III knee osteoarthritis. However, the lack of homogeneity in the included studies and the variance in MSCs sources and preparations should be noted.

**Level of evidence:**

III.

## Introduction

Pain and functional limitations associated with knee osteoarthritis (OA) are frequent and negatively influence patients' quality of life [11]. Available conservative treatments include, among others, intraarticular injections of corticosteroids, hyaluronic acid (HA), blood derivatives products such as platelet-rich plasma (PRP), or the administration of human cells, known as cell therapy (CT). Indeed, cell therapy has gained more attention in recent decades and features an increasingly accepted treatment modality for knee OA [18].

Mesenchymal stem cells (MSCs) hold a great place in this direction and play a crucial role in tissue homeostasis, repair, and regeneration. Mesenchymal stem cells are characterized by their potential for self-renewal, plasticity, and the aptitude to differentiate into specific tissue cell types, including cartilage and bone cells [23]. More specifically, stem cells could act through different mechanisms, decreasing inflammation, recruiting cells, regulating the immune response, reducing apoptosis, and stimulating angiogenesis [17].

The scientific literature demonstrated growing evidence of efficacy in pain relief and increased function for chondral defects and OA using MSCs [19]. There are different sources of autologous MSCs in clinical practice; they are obtained most often from the bone marrow in the form of bone marrow aspirate concentrate (BMAC) or from the adipose tissue as a stromal vascular fraction (SVF) [15, 31]. Some authors used the same sources after culture expansion to achieve a higher number of stem cells again with promising results either as an injectable treatment or after surgical implantation [5, 12–14].

Several studies have reported decreased pain and improved functional scores after stem cell administration through intraarticular injection in cases of advanced OA [15]. However, the evidence of this challenging topic is constantly increasing and changing. This research systematically reviews the literature for recent studies focusing on autologous minimally processed MSCs administrated with a single intraarticular injection for early to moderate knee OA. It aims to expose post-injection patient-reported outcome measures (PROMs), radiological evaluation, and complications and give an overall use.

## Materials and methods

The present systematic review was conducted following the Preferred Reporting Items for Systematic Review and Meta-Analysis (PRISMA) guidelines [16].

### Search strategy

A comprehensive literature search was performed, by two investigators (Τ.Κ., C.P.), using the Medline (PubMed), Web of Science, Scopus, and Cochrane Central databases on March 2023. Firstly, the titles and abstracts of the identified studies were assessed for eligibility, followed by the full-text articles screening. Moreover, the references of the included articles were screened. A third researcher (M.I.) helped resolve any disagreement and provide a consensus.

### Eligibility criteria

All studies that satisfied the following criteria were included in the present systematic review: i) studies on adult human subjects treated with intra-articular injections of MSCs recording PROMS, ii) OA classified as Kellgren-Lawrence I-IIΙ, iii) single injection treatment, iv) peer-reviewed articles published within the last six years and v) English language published studies.

The exclusion criteria were: i) studies used culture-expanded MSCs, ii) studies performed on cartilage defects, iii) intra-articular injections in combination with surgical procedures, iv) studies reporting incomplete data, and v) abstract, review articles, meta-analyses, or in vitro studies/animal studies.

### Level of evidence and quality of studies

Each study's evidence level was assessed using the modified criteria of the Oxford Centre for Evidence‐Based Medicine Working Group (OCEBM). The studies were qualitatively assessed using the revised and validated version of the MINORS (Methodological Index for Non-Randomized Studies) score for the non-randomized studies and the MJS (Modified Jaded Scale) for the randomized control trials.

### Data extraction and analysis

Data were extracted and recorded from each study as follows: first author, year of publication, study type, age, gender, sample size (number of patients and/or number of knees), grade of OA, MSCs quantity and source, Body Mass Index (B.M.I.), mean follow-up. The collected outcome measures that were available consisted of the Visual Analogue Scale (VAS) for pain, the Western Ontario Macmaster University Osteoarthritis Index (W.O.M.A.C.), the Knee Injury and Osteoarthritis Outcome Score (K.O.O.S), the Knee Society Score (K.S.S.) clinical and functional, the Lysholm Knee Scoring Scale, the Japanese Knee Osteoarthritis Measure (JKOM), the Numeric Rating Scale (NAS), the EQ-5D-5L questionnaire, complications, and radiographic analysis. A pooled analysis and a meta-analysis of the clinical outcomes were considered inappropriate due to the heterogeneity of the included studies and the significant risk of bias.

## Results

### Literature search and study identification

**Fig. 1:**
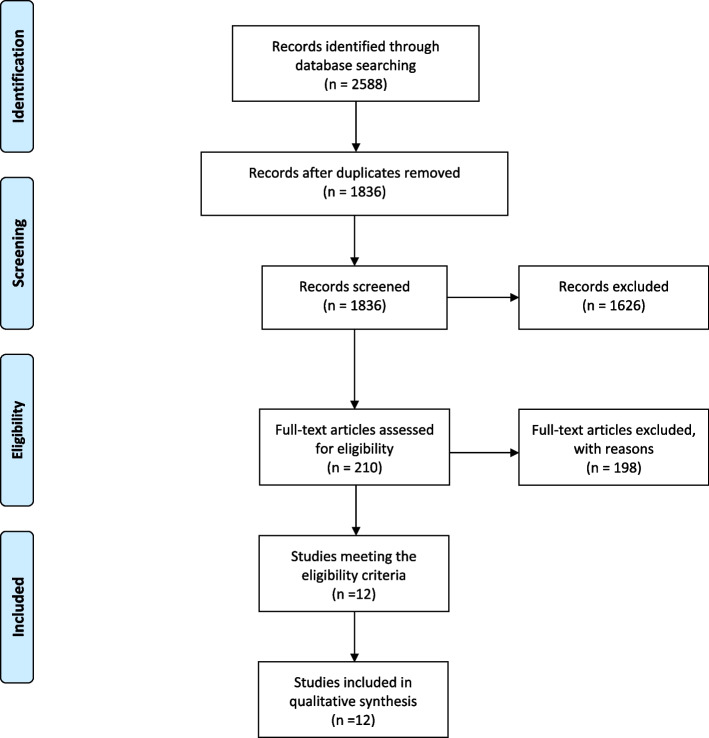
PRISMA flow diagram for search results. From:  Moher D, Liberati A, Tetzlaff J, Altman DG, The PRISMA Group (2009). Preferred Reporting Items for Systematic Reviews and Meta-Analyses: The PRISMA Statement. PLoS Med 6(6): e1000097. doi:10.1371/journal.pmed1000097

The literature search yielded 2558 potentially relevant studies. A total of 722 duplicate articles were excluded; thus, after the removal, 1836 were identified. The screening of the titles and abstracts demonstrated 1626 irrelevant articles. Two hundred ten records were retrieved for full-text evaluation. Based on inclusion and exclusion criteria, a further 198 articles were excluded. The remaining 12 studies were eligible for inclusion. The PRISMA flowchart (Fig. 1) illustrates the whole selection process.

### Characteristics of the eligible studies and patients

A total of 539 patients and 576 knees treated with intraarticular injection of stem cells were included in this systematic review. The mean reported age in the included studies ranged from 54—73 years, and the mean B.M.I. from 25.1 – 27 kg/m. The follow-up ranged from 3 months to at least 5 years. All the included studies were level 1 or 2. Characteristics of the included studies, study period, type of the study, level of evidence, quality of studies, and patient demographics are summarised in Table 1. The risk of bias in the analysed studies was low based on the MINORS (range 12/16—14/16) and the MJS (range 5/8 – 8/8).

**Table 1:** Level of evidence (LoE), Quality of Study and demographics of patients included in the 12 studies

Author	Study Type	LoE	Quality of study	N patients (Knees)	Lost FU	MSCs	Control Group	Gender (M/F)	Mean Age (years)	BMI (kg/m2)	Mean FU (months)	FU (range)	Study Period
Garay-Mendoza 2017 [7]	PCS	3	14/16	30 (30)	4	BMAC	Acetaminophen (31)	7/23	55.7	29.5	6		n.a
Tsubosaka 2020 [21]	PCoS	3	13/16	60 (60)	3	SVF		n.a	69.4	25.1	13.7	11.7–15.7	September 2017 – March 2018
Wells 2020 [24]	PCoS	3	13/16	10 (13)		BMAC		4/6	55.3	n.a	12		April 2017—December 2018
Varady 2020 [22]	PCoS	3	12/16	17 (17)		BMAC		7/10	54	n.a	12		March 2018—March 2019
Estrada 2020 [3]	PCS	3	13/16	33 (33)		SVF	PRP (29)	n.a	54.7	25.5	12		March 2012—July 2019
				27 (27)		BMAC		n.a	54.7	25.5	12		
Garza 2020 [8]	RCT	2	7/8	13 (13)		SVF	Placebo	4/9	60.5	27.6	12		July 2016 – September 2017
				13 (13)		SVF DD	Placebo	7/6	59.5	28.8	12		
Kaszynski 2022 [10]	RCT	2	6/8	20 (20)		SVF	PRP (20) Control (20)		55	27	12		n.a
Yokota 2022 [27]	POC	3	14/16	38 (69)		SVF	Culture ASC (72)	7/31	73	25	24		November 2016 – April 2017
Zhang 2022 [28] (Stem Cell Research & Therapy)	RCT	2	8/8	56 (56)	5	SVF	HA (64)	14/42	54	23.7	60		May 2013—July 2015
Zhang 2022 [29] (BioMed Research International)	RCT	2	6/8	50 (53)		SVF	HA (51)	18/29	50.8	22.7	12		January 2018—May 2021
Altetto 2022 [1]	PCoS	3	12/16	123 (123)		SVF		57/66	57	27	6		June 2019—November 2020
Anz 2022 [2]	RCT	3	5/8	49 (49)	4	BMAC	PRP (39)	27/18	55.8	27.7	24		n.a

*BMAC* Bone Marrow Aspirate Concentrate, *SVF* Stromal Vascular Fraction. *SVF DD* Stromal Vascular Fraction Double Dose, *PCS* Prospective Comparative Study, *PCoS* Prospective Cohort Study, *RCT* Randomised Control Trial, *POC* Parallel Observation Cohort, *ASC* Adipose Stromal Cells

### MSCs sources and site of injection

All the included studies used autologous-derived MSCs. Seven studies used MSCs provided by the abdominal adipose tissue [1, 10, 21, 27–29], four used bone marrow from the iliac crest [2, 7, 22, 24], and one from both sources [3]. Different reported devices were used for harvesting adipose or bone marrow-derived MSCs (Celution, Lipogems, Lipocell, GID SVF-2, PureBMC) [2, 8, 10, 21, 27, 28]. The most often superolateral approach was used, and some authors injected [2, 21, 22, 24] it under ultrasound guidance. Cell number was calculated in four studies [8, 21, 24, 28].

### Clinical outcomes

Table 2 summarizes all reported outcomes that were improved in all eligible studies. Most studies evaluated pain relief with the VAS score [1, 7, 10, 21, 22, 27, 29]. Several PROMs were used to quantify clinical outcomes. Six studies reported the Western Ontario Macmaster University Osteoarthritis Index (WOMAC) [2, 7, 8, 10, 21, 28], five the Knee Injury and Osteoarthritis Score (KOOS) [1, 10, 21, 22, 27], two the International Knee Documentation Committee (IKDC) [3, 10], and from one study the Knee Society Score (KSS) clinical and functional [3], the Japanese Knee Osteoarthritis Measure (JKOM) [21], and the Lysholm score [22]. One study used the EQ-5D-5L to assess health-related quality of life. The same research estimates patients' mobility with the time up and go, five times sit to stand, and 10 min walk tests [10].

**Table 2:** A summary of the mean outcome scores from the various publications

Score	Author	Year	Number of knees	Modality	K-L Grade	Pre-injection score	Post-injection score	Improvement points	Time of post injection score	Notes (*p*-values)
VAS	Garay-Mandoza [7]	2017	26	BMAC	2–3	5.3	0.9	4.4	6 months	Improved (n.a.)
	Tsubosaka [21]	2020	11	SVF	2	3.6	2.1	1.5	12 months	Improved (n.a.)
	Tsubosaka [21]	2020	36	SVF	3	5.2	3.7	1.5	12 months	Improved (n.a.)
	Varady [22]	2020	17	BMAC	1–3	2.7	2.1	0.6	3 months	Improved (*p* = 0.003)
	Yokota [27]	2022	30	SVF	2–3	7.2	5	2.2	2 years	Improved (*p* < 0.05)
	Kaszynski [10]	2022	20	AAT	2–3	5.1	2.8	2.3	12 months	Improved (*p* < 0.001)
	Aletto [1]	2022	123	SVF	1–3	6.5	2	4.5	6 months	Improved (*p* < 0.05)
	Zhang [28] (Stem Cell Research & Therapy)	2022	56	SVF	2–3	4	1.8	2.2	5 years	Improved (*p* < 0.001)
	Zhang [29] (BioMed Research International)	2022	29	SVF	2	4.3	0.9	3.4	12 months	Improved (*p* < 0.001)
	Zhang [29] (BioMed Research International)	2022	24	SVF	3	6	0.8	5.2	12 months	Improved (*p* < 0.001)
WOMAC	Garay-Mandoza [7]	2017	26	BMAC	2–3	62.6	91.7	30.1	6 months	Improved (n.a.)
	Tsubosaka [21]	2020	11	SVF	2	27.7	11.2	16.5	12 months	Improved (n.a.)
	Tsubosaka [21]	2020	36	SVF	3	35.1	26	9.1	12 months	Improved (n.a.)
	Garza [8]	2020	13	SVF	2–3	56.2	21.8	34.4	12 months	Improved (n.a.)
	Garza [8]	2020	13	SVFx2	2–3	47.1	13.2	33.9	12 months	Improved (n.a.)
	Anz [2]	2022	45	BMAC	1–3	35.3	20.8	14.5	2 years	Improved (*p* < 0.01)
	Kaszynski [10]	2022	20	AAT	2–3	63.9	83.6	19.7	12 months	Improved (*p* < 0.001)
	Zhang [28] (Stem Cell Research & Therapy)	2022	56	SVF	2–3	33.2	27	6.2	5 years	Improved (*p* < 0.001)
WOMAC Pain	Anz [2]	2022	45	BMAC	1–3	7	3.8	3.2	2 years	Improved (*p* < 0.01)
	Zhang [29] (BioMed Research International)	2022	29	SVF	2	9,4	2,7	6,7	12 months	Improved (*p* < 0.001)
WOMAC Physical Function	Anz [2]	2022	45	BMAC	1–3	22.9	13.2	9.7	2 years	Improved (*p* < 0.01)
	Zhang [29] (BioMed Research International)	2022	29	SVF	2	24.7	10.1	14.6	12 months	Improved (*p* < 0.001)
WOMAC Stiffness	Anz [2]	2022	45	BMAC	1–3	3.8	2.2	1.6	2 years	Improved (*p* < 0.01)
	Zhang [29] (BioMed Research International)		29	SVF	2	2.8	0.9	1.9	12 months	Improved (*p* < 0.001)
KOOS	Tsubosaka [21]	2020	11	SVF	2	52.9	68.5	15.6	12 months	Improved (n.a.)
	Tsubosaka [21]	2020	36	SVF	3	48.6	56.3	7.7	12 months	Improved (n.a.)
	Aletto [1]	2022	52	SVF	1	55.4	89.8	34.4	6 months	Improved (*p* < 0.05)
	Aletto [1]	2022	61	SVF	2	49.9	85.9	36	6 months	Improved (*p* < 0.05)
	Aletto [1]	2022	10	SVF	3	45	84.1	39.1	6 months	Improved (*p* < 0.05)
	Yokota [27]	2022	30	SVF	2–3	40	56	16	2 years	Improved (*p* < 0.05)
KOOS pain	Varady [22]	2020	17	BMAC	1–3	53.8	83	29.2	3 months	Improved *p* < 0.001
	Kaszynski [10]	2022	20	AAT	2–3	57.8	78.9	21.1	12 months	Improved (*p* < 0.001)
KOOS Symptoms	Varady [22]	2020	17	BMAC	1–3	50.9	74.5	23.6	3 months	Improved (*p* = 0.053)
	Kaszynski [10]	2022	20	AAT	2–3	57.7	78.9	21.2	12 months	Improved (*p* < 0.001)
KOOS ADL	Varady [22]	2020	17	BMAC	1–3	61.1	89.3	28.1	3 months	Improved *p* < 0.001
	Kaszynski [10]	2022	20	AAT	2–3	63.7	84	20.3	12 months	Improved (*p* < 0.001)
KOOS Sports/Rec	Varady [22]	2020	17	BMAC	1–3	36.9	72.6	35.7	3 months	Improved (*p* = 0.006)
	Kaszynski [10]	2022	20	AAT	2–3	35.5	66.1	30.6	12 months	Improved (*p* < 0.001)
KOOS QOL	Varady [22]	2020	17	BMAC	1–3	32.7	66.1	33.4	3 months	Improved (*p* = 0.003)
	Kaszynski [10]	2022	20	AAT	2–3	38.8	62.2	23.4	12 months	Improved (*p* < 0.001)
KOOS Index	Kaszynski [10]	2022	20	AAT	2–3	55.7	77.9	22.2	12 months	Improved (*p* < 0.001)
KOOS Joint Replacement	Wells [24]	2020	13	BMAC	1–2	63.1	80.3	17.2	12 months	Improved (*p* < 0.001)
IKDC	Kaszynski [10]	2022	20	AAT	2–3	44.2	68.9	24.7	12 months	Improved (*p* < 0.001)
	Estrada [3]	2020	33	SVF	3	33.9	64.2	30.3	12 months	Improved (n.a.)
	Estrada [3]	2020	27	BMAC	3	30.2	57.6	27.4	12 months	Improved (n.a.)
Lysholm	Varady [22]	2020	17	BMAC	1–3	55.5	77.3	21.8	3 months	Improved *p* = 0.009
KSS-C	Estrada [3]	2020	33	SVF	3	38.9	65.6	26.7	12 months	Improved (n.a.)
	Estrada [3]	2020	27	BMAC	3	33.8	56.7	22.9	12 months	Improved (n.a.)
KSS-F	Estrada [3]	2020	33	SVF	3	53.3	76.7	23.4	12 months	Improved (n.a.)
	Estrada [3]	2020	27	BMAC	3	52	75.6	23.6	12 months	Improved (n.a.)
JKOM	Tsubosaka [21]	2020	11	SVF	2	27.4	11.4	16	12 months	Improved (n.a.)
	Tsubosaka [21]	2020	36	SVF	3	37.4	31.6	5.8	12 months	Improved (n.a.)
EQ-5D-5L	Kaszynski [10]	2022	20	AAT	2–3	70	80	10	12 months	Improved *p* < 0.01
Time Up & Go (sec)	Kaszynski [10]	2022	20	AAT	2–3	6.9	5.6	1.3	12 months	Improved (*p* < 0.001)
5 Times Sit to Stand (sec)	Kaszynski [10]	2022	20	AAT	2–3	10.8	8.2	2.6	12 months	Improved (*p* < 0.001)
10 min walk test	Kaszynski [10]	2022	20	AAT	2–3	6.2	4.8	1.4	12 months	Improved (*p* < 0.001)

Referred to data published in *VAS* Visual Analog Scale for pain, *WOMAC* Western Ontario Macmaster University Osteoarthritis Index, *IKDC* International Knee Documentation Committee, *KOOS* Knee Injury and Osteoarthritis Score, *KSS-C* Knee Society Score-Clinical, *KSS-F* Knee Society Score-Functional, *JKOM* Japanese Knee Osteoarthritis Measure, *n.a.* not applicable

### Radiological outcomes

Two studies analyzed radiological changes [28, 29]. One study used X-rays to determine the KL grade and the mechanical axis of the knee and MRI to evaluate the cartilage structure and volume, patella-femoral pathology, and bone marrow lesions and compared the pre-injection and 5-year follow-up status [28]. Based on the X-rays, the KL grade was increased by 15.7% and remained unchanged at 84.3%, and the varus mechanical axis was increased from 1,5° to 1,8^ο^. The MRI findings were as follows: the size of the full-thickness defect decreased by 5.9%, increased by 7.8%, and remained unchanged by 86.3%; the total cartilage volume decreased from 16.467,1 mm3 to 15.121,1 mm^3^; the rate of patella-femoral degeneration was increased from 49% to 58.8%; and the bone marrow lesion size was decreased from 123,5 mm^2^ to 90,3 mm^2^. The other study used the Whole-Organ Magnetic Resonance Imaging Score (WORMS) to assess the knee, while the MOCART score was used to examine cartilage repair [29]. Between the baseline and the 12-month follow-up, the WORMS improved from 54.9 to 40.5 in the KL2 group and from 75.7 to 57.5 in the KL3 group. Between the 6- and 12-month evaluation, the MOCART score was improved from 52.9 to 62.1 in patients with KL2 and from 46.5 to 57.1 in patients with KL3.

### Complications

In total, three studies reported complications. One of them noted weakness in the knee (2 knees), pain around the injection site (1 knee), minor bleeding from the aspiration site (1 knee), and minor redness or swelling around the injection site (3 knees) [24]. The two other post-injection swelling in one knee [2, 7].

## Discussion

The most important finding of this systematic review was that a single intraarticular administration of MSCs is a safe and efficient treatment option with good clinical results for dealing with early to moderate knee joint OA.

The recent literature has demonstrated encouraging results in managing knee OA. For instance, Song et al. [20], in a meta-analysis of 15 randomized control trials, including 584 patients with knee OA, found that the injection of MSCs has been associated with a significant decrease in both VAS and WOMAC scores at 12 months and six months follow-up, respectively, compared to controls. In another most recent meta-analysis of 43 studies, Zhao et al. [30] found an improvement in pain and functional scores at six but not 12 months after MSCs injection. Similarly, Shoukrie et al. [19] systematically reviewed ten studies. They concluded that MSCs injection significantly improved VAS, WOMAC, and KOOS scores and featured better post-injection MRI findings in patients with osteoarthritic knees. However, this study is differentiated from the present systematic review as it includes all grades of K-L classification.

Nevertheless, some authors question such studies' reliability and risk of bias [6, 25, 26]. In light of the above hesitation, the present systematic review tries to cover this question and contributes to the evidence of this challenging topic. This systematic review included 12 high-level evidence studies, treating 539 patients and 576 knees with a single intraarticular injection of MSCs. The reported outcomes showed that intraarticular administration of MSCs is an efficient and safe procedure associated with reduced pain and increased function in patients with early to moderate knee OA.

The VAS was mainly used to evaluate pain relief, and eight studies [1, 7, 10, 21, 22, 27–29] assessed it at baseline and after administration. The follow-up period was ranged between 3 months to 5 years, and the improvement varied between 0.9 to 5.2 points. It is essential to note that all the studies included patients with KL grade 1 to 3 OA, but only two studies [21, 29] presented the results of each degree separately. Thus, evaluating the improvement and efficacy in different severities of knee OA is difficult.

On the other hand, six studies [2, 7, 8, 10, 21, 28] described the clinical evaluation of the patients before and after the administration of the MSCs with the WOMAC score. One study [7] had a limited follow-up period of 6 months, three studies [8, 10, 21] reported the results after 12 months, and two studies had more than one-year evaluation, precisely 2 [2] and 5 [28] years, respectively. Again, all analyses presented an improvement. However, almost all studies have shown short-term clinical outcomes, and thus the interpretation of these results should be made cautiously. One of the studies showed the K-L grade 2 and 3 OA results separately and demonstrated more remarkable progress in the case of grade 2 arthritis which was predictable [21]. Another study [8] reported the results in two groups of thirteen patients receiving low and high doses of SVF and reported a dose-dependent improvement with the high-dose group to present better results. Again, these results are unsurprising as the literature has already demonstrated a clear relationship between the number of MSCs and better outcomes [9].

Two included studies were randomized control trials comparing the outcomes after SVF administration to hyaluronic acid. Zhang et al. [28] concluded that VAS and WOMAC scores in the SVF group were significantly better than in the HA group during the 5-year follow-up after treatment. Moreover, the average responsive time to SVF treatment (61.5 months) was significantly longer than the HA treatment (30.4 months) calculated by the Kaplan–Meier responsive curves. The other study [29] compared 53 knees with K-L grade 2 and 3 OA that received an intra-articular injection of SVF and 51 knees that received HA. The patient's VAS, WOMAC pain, stiffness, and physical function were evaluated at baseline and 1, 3, 6, and 12 months after injection with SVF and HA. SVF-treated knees showed significant improvement in clinical scores at both grades of OA till the final follow-up. On the contrary, in the control group, clinical scores were relieved one month after HA injection and amplified until the 12-month follow-up evaluation. Therefore, both studies demonstrated that using MSCs provides better clinical outcomes than HA. Moreover, it is a safe procedure as minor, or no adverse events were recorded in these studies.

In the last few years, there has also been a constantly increasing interest in platelet-rich plasma (PRP) injections. The PRP is derived after autologous blood centrifugation and contains several growth factors to cope with musculoskeletal disorders, mainly knee OA [4]. This systematic review includes two randomized control trials comparing the clinical outcomes of MSCs to that of platelet-rich plasma. Interestingly enough, both studies do not demonstrate the superiority of MSCs on PRP.

More precisely, Anz et al. [2] compared the efficacy of BMAC and PRP on pain and function in patients with knee OA up to 24 months after injection. Ninety symptomatic participants (KL grades 1–3) were randomized into PRP and BMAC injection groups. Both groups completed the WOMAC and subjective IKDC questionnaire before and after a single intra-articular leukocyte-rich PRP or BMAC injection. Both groups had significantly improved from baseline to 24 months after the injection; however, no difference was found at any time during the evaluation. In the other study, Estrada et al. [3] compared PRP, BMAC, and adipose-derived MSCs injections in treating OA of the knee using functional scores. Again, a statistically significant improvement was observed in the three groups at all time points during follow-up compared with baseline; nevertheless, without difference among treatment types.

The strengths of this review are the following: first, the fact of reporting the outcomes following intraarticular injection of autologous MSCs in early to moderate OA. Second, focusing on the recent literature as the included studies are published within the last six years. Third, all the included studies are of a high level of evidence. The clinical relevance of the present study is that it provides evidence to endorse the use of MSCs intraarticular injection for knee OA. Based on the promising outcomes, there should be part of the possible treatment algorithm during the decision-making in patients suffering from early to moderate knee OA.

This study should be considered on the subject of the following limitations. Firstly, the included studies lack homogeneity regarding PROMs, and thus the study was limited to presenting the data descriptively. Moreover, the diversity in the included studies and the variance in MSCs sources, preparations, and administration make the treatment not reproducible. Next, most of the included studies had a limited number of participants with different grades of O.A. Therefore, evaluating the treatment's effectiveness in each stage of O.A. is ambitious. Another potential limitation is that most included studies referred to a relatively short follow-up period. Nevertheless, ten of the twelve included studies had at least twelve months of follow-up, which is considered a sufficient period to evaluate the treatment's efficacy in this kind of research. However, further studies with a higher follow-up time are needed to validate these results. Last, the number of stem cells applied was not quantified to verify the required quantity to reach positive results. However, this is a common limitation in similar studies, as quantification of the cells is not routinely performed during the procedure.

## Conclusion

Based on high-level evidence studies, the single intra-articular injection of MSCs is a safe, reliable, and effective treatment option for Kellgren-Lawrence grade I—III knee OA patients However, the lack of homogeneity in the included studies and the variance in MSCs sources and preparation should be noted.

## Abbreviations

BMAC: Bone Marrow Aspirate Concentrate
BMI: Body Mass Index
CT: Cell Therapy
HA: Hyaluronic Acid
IKDC: International Knee Documentation Committee
JKOM: Japanese Knee Osteoarthritis Measure
KL: Kellgren-Lawrence
KOOS: Knee injury and Osteoarthritis Outcome Score
KSS: Knee Society Score
MINORS: Methodological Index for Non-Randomized Studies
MJS: Modified Jaded Scale
MOCART: Magnetic Resonance Observation of Cartilage Repair Tissue
MSCs: Mesenchymal Stem Cells
NAS: Numeric Rating Scale
OA: Osteoarthritis
OCEBM: Oxford Centre for Evidence‐Based Medicine Working Group
PRISMA: Preferred Reporting Items for Systematic Review and Meta-Analysis
PROMs: Patient Reported Outcomes Measures
PRP: Platelet-Rich-Plasma
SVF: Stromal Vascular Fraction
VAS: Visual Analogue Scale
WOMAC: Western Ontario Macmaster University Osteoarthritis Index
WORMS: Whole-Organ Magnetic Resonance Imaging Score
